# Development and Field Validation of a Smartphone-Based Web Application for Diagnosing Optimal Timing of Mid-Season Drainage in Rice Cultivation via Canopy Image-Derived Tiller Estimation

**DOI:** 10.3390/s26031000

**Published:** 2026-02-03

**Authors:** Yusaku Aoki, Atsushi Mochizuki, Mitsuaki Nakamura, Chikara Kuwata

**Affiliations:** Chiba Prefectural Agriculture and Forestry Research Center, Chiba Prefectural Office, 180-1 Ookanazawa-Cho, Midori-Ku, Chiba-City 266-0014, Japan

**Keywords:** smartphone imaging, rice (*Oryza sativa*), tiller estimation, mid-season drainage, phenological decision support, image-based diagnosis, web application, smart agriculture

## Abstract

In recent years, excessive tillering caused by high temperatures during early growth has contributed to rice quality deterioration in warm regions of Japan. Accurate determination of midseason drainage timing is essential but remains difficult due to year- and cultivar-dependent variability. In this study, we developed a smartphone-based web application that estimates rice tiller number from canopy images and diagnoses the optimal timing of midseason drainage by comparing estimated tiller numbers with cultivar-specific target values. The system operates entirely on a smartphone using HTML5 canvas-based pixel extraction, JavaScript computation, and Google Apps Script-based backend processing. Field experiments conducted in Chiba Prefecture using three rice cultivars showed a strong linear relationship between estimated and observed tiller numbers (R^2^ = 0.9439). The root mean square error (RMSE) was 42.6 tillers m^−2^, with a consistent negative bias (−34.6 tillers m^−2^), indicating systematic underestimation. Considering typical tiller increase rates near midseason drainage (12.0–24.3 tillers m^−2^ day^−1^), these errors correspond to approximately 1–3 days of growth progression, which is acceptable for timing-based decision-making. Although the system does not aim to provide precise absolute tiller counts, it reliably captures relative growth-stage dynamics and supports threshold-based diagnosis. The proposed approach enables rapid, on-site decision support using only a smartphone, contributing to labor-saving and improved water management in rice production.

## 1. Introduction

Rice (*Oryza sativa* L.) is one of the world’s most important crops, providing food for more than half of the global population [1]. In recent years, increases in mean air temperature and the frequency of extreme high-temperature events in major rice-growing regions have been widely reported as consequences of global climate change [2,3]. In Japan, high temperatures during the early growth stage of rice have become a serious problem, causing excessive vegetative growth [4,5].

Rice tiller number is determined by complex interactions among multiple factors, including cultivar traits, nitrogen management, planting density, solar radiation, soil fertility, and water management. In warm-region early-season rice cultivation, elevated temperatures immediately after transplanting have recently become a major driver of accelerated vegetative growth and excessive tillering.

In Chiba Prefecture, which practices early-season rice cultivation in a warm climate, the average temperature during the rice-growing season (April–August) has shown an increasing trend, with particularly high temperatures frequently observed in May [6,7]. In this region, April 20 is the standard transplanting date, and elevated temperatures immediately after transplanting accelerate early vegetative growth and increase tiller number. As a result, excessive spikelet formation often occurs, leading to deterioration in grain appearance quality and a higher risk of lodging [8,9]. Moreover, the recent increase in extreme high-temperature days has made it increasingly difficult to achieve management targets such as the “target tiller number” in some years [10].

Globally, excessive vegetative growth during the early growth stage is recognized as a common problem in major rice-producing regions, and the proper implementation of water management according to growth stage is essential for stabilizing yield [11]. Under such conditions, midseason drainage has become a key practice for regulating tiller number during the early growth stage. Midseason drainage is known to suppress excessive vegetative growth, reduce lodging risk, and improve root-zone conditions [12]. Physiologically, midseason drainage regulates tiller development through moderate water stress, which suppresses excessive vegetative growth and the emergence of late tillers. Improved root-zone aeration enhances root activity and nutrient uptake efficiency, while assimilate allocation shifts from vegetative organs toward developing panicles, contributing to a more balanced source–sink relationship. This practice also contributes to the reduction in methane emissions from paddy fields and has attracted attention as an effective agricultural practice compatible with the Sustainable Development Goals [13,14]. However, determining the optimal timing of midseason drainage remains difficult, as early implementation leads to an insufficient panicle number, whereas delayed implementation causes excessive growth and the occurrence of late-developing panicles [15]. Furthermore, the recent shortage of skilled farmers has intensified the demand for objective and reproducible technologies to support such management decisions.

The major rice cultivars cultivated in Chiba Prefecture, namely ‘Fusaotome’, ‘Fusakogane’, and ‘Koshihikari’, differ in their growth characteristics and plant architecture [16]. ‘Fusaotome’ is an early-maturing, panicle number-driven cultivar, for which securing a sufficient number of tillers is particularly important for yield formation. In contrast, ‘Fusakogane’ is a medium-maturing cultivar and ‘Koshihikari’ is a late-maturing cultivar; both exhibit intermediate growth characteristics between panicle number-driven and panicle weight-driven cultivars. As a result, appropriate tiller number management is required to avoid excessive vegetative growth while maintaining yield potential. Based on these cultivar-specific growth characteristics, Chiba Prefecture has established optimal target tiller numbers according to cultivar and soil type [17]. Consequently, optimized management decisions based on cultivar-specific target tiller numbers (diagnostic scores in this study) are essential for ensuring grain quality. However, manual measurement of tiller number is labor-intensive and impractical when applied to multiple fields.

With the promotion of smart agriculture, cultivation support systems using ICT and sensor technologies have been increasingly adopted worldwide [18,19,20,21]. Image-based diagnostic technologies using UAV and satellite imagery have demonstrated high effectiveness in estimating canopy characteristics such as leaf area index, aboveground biomass, and yield [22,23,24,25]. However, high equipment costs and the requirement for specialized analytical skills limit their practical use by small-scale farmers [26,27].

In contrast, smartphones are equipped with high-resolution cameras and offer low cost and high portability, and their application to rice canopy structure analysis and growth diagnosis is rapidly expanding [28,29,30]. Recent studies have also reported panicle detection and growth stage recognition using smartphone-based RGB images [31]. Nevertheless, many of these studies rely on indirect growth indicators such as canopy cover and leaf color [32], and technologies that directly estimate tiller number and support practical water management decisions remain under development.

Although these advances are notable, there is currently no operational system that enables farmers to directly estimate rice tiller number using only a smartphone and immediately link this information to practical water-management decisions. Most existing approaches require UAVs, satellites, or PC-based post-processing, which limits real-time, on-site usability. Therefore, a clear scientific and technological gap exists in the development of a lightweight, smartphone-only tool capable of real-time tiller estimation and threshold-based diagnosis of optimal midseason drainage timing under field conditions.

In Chiba Prefecture, early attempts to estimate tiller number from smartphone images required PC-based analysis, which limited on-site operability [33,34]. Subsequently, web-based estimation technologies were developed; however, their estimation accuracy and cultivar-dependent differences were not sufficiently validated [35,36].

Access to timely and objective estimates of rice tiller number can directly contribute to improved crop management and farm profitability. Accurate assessment of tiller dynamics enables farmers to optimize midseason drainage timing, thereby avoiding both insufficient panicle formation caused by premature drainage and excessive vegetative growth resulting from delayed drainage. This supports stable yield formation, improved grain quality, and reduced lodging risk. Moreover, timely tiller-based decision-making can reduce unnecessary water use, fertilizer inputs, and labor associated with corrective field operations, contributing to lower production costs. For these reasons, a smartphone-based tiller estimation tool has strong potential to enhance both agronomic efficiency and economic sustainability in rice production systems.

In this study, we developed a smartphone-based web application that estimates rice tiller number from canopy images and diagnoses the optimal timing of midseason drainage by comparing the estimated tiller number with cultivar-specific target values. Furthermore, we evaluated the estimation accuracy, operability, and practical applicability of the proposed system through field experiments conducted in paddy fields in Chiba Prefecture.

Although the core tiller estimation algorithm builds on our previous work, the novelty of the present study lies in the engineering integration of smartphone-based image sensing, real-time on-site processing, and threshold-based agronomic decision support. Specifically, this study establishes an operational framework in which image-derived tiller dynamics are directly translated into actionable guidance for midseason drainage timing under practical field conditions.

## 2. Materials and Methods

### 2.1. Overview of the Diagnostic System

The midseason drainage diagnostic system developed in this study (hereafter referred to as “the proposed system”) is a web-based application that estimates tiller number from rice canopy images captured using a smartphone and determines the optimal timing of midseason drainage by comparing the estimated tiller number with cultivar-specific target values.

The proposed system consists of a series of processing steps, including image acquisition, RGB-based pixel analysis, calculation of canopy cover ratio and estimated tiller number, comparison with cultivar-specific threshold values, and display of the diagnostic result.

Users first select the target rice cultivar and upload an image of the paddy field captured with a smartphone to the application. The uploaded image is automatically analyzed, and the canopy cover ratio and estimated tiller number are calculated based on the proportion of extracted rice leaf and stem regions. The estimated tiller number is then compared with the cultivar-specific target value. If the estimated value reaches the target level, the system diagnoses the field as being at the “optimal timing for midseason drainage”; otherwise, it is diagnosed as having an “insufficient tiller number”. The diagnostic result is displayed immediately on the application screen, thereby enabling rapid on-site decision-making in practical rice production.

**Figure 1:**
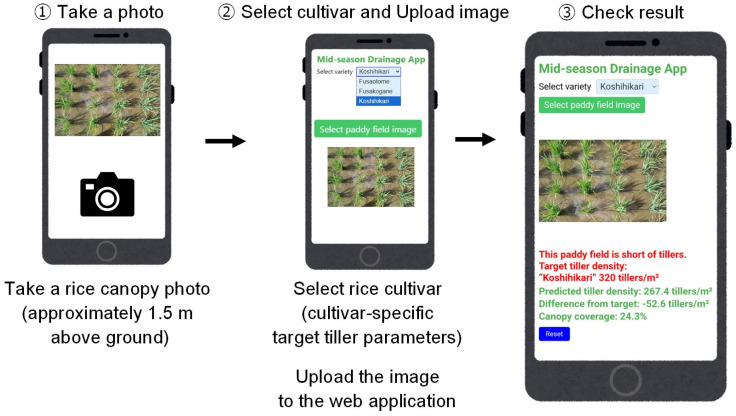
Operational workflow of the smartphone-based midseason drainage diagnosis system. The workflow consists of three steps: (**1**) acquisition of a rice canopy image using a smartphone at approximately 1.5 m above ground, (**2**) selection of the rice cultivar to set cultivar-specific target tiller parameters and upload of the captured image to the web application, and (**3**) on-screen display of the estimated tiller number, canopy cover, and midseason drainage diagnosis result.

Figure 1 illustrates the basic operational workflow of the diagnostic system developed in this study. Users can complete the entire procedure—from image acquisition to confirmation of the diagnostic results—step by step through simple operations, without requiring any specialized settings or technical expertise.

### 2.2. Design and Implementation of the Smartphone-Based Web Application

#### 2.2.1. Technologies and System Architecture

The proposed system was developed as a web-based application that operates on a smartphone web browser. HTML5, JavaScript, and Google Apps Script (GAS; V8 runtime environment) were used for application development. As these technologies are continuously updated, the system was developed and tested using the stable web environment available in 2024. For image processing, the HTML5 canvas element was employed, and pixel-level data analysis was performed using JavaScript. Part of the diagnostic decision processing was executed on the server side using GAS.

GAS was adopted as the backend platform for the following reasons. First, GAS is a free serverless execution environment provided by Google and does not require the construction or maintenance of a dedicated server, thereby enabling stable operation while minimizing operational costs. Second, GAS allows backend processing for web applications to be implemented easily, which reduces both development complexity and maintenance workload. Based on these advantages, GAS was considered a suitable development platform for small-scale smart agriculture support applications such as the system proposed in this study.

An overview of the system requirements and processing time is summarized in Table 1. The proposed system operates on a standard web browser installed on an Android smartphone, and image analysis is executed as an online process. The time required from image upload to on-screen display of the diagnostic result was approximately 5 s.

As a result, the proposed system does not require any software installation and can be accessed directly through a standard smartphone web browser. This configuration enables real-time, on-site diagnosis in paddy fields using only a smartphone.

#### 2.2.2. Image Analysis and Tiller Number Estimation Algorithm

The images used for analysis were RGB color images of the rice canopy captured vertically from above using a smartphone camera (8-bit, 256 grayscale levels). The captured images were loaded onto the HTML5 canvas, and the RGB values of each pixel were obtained. For the extraction of rice leaf and stem regions, a discriminant value I was calculated based on the following equation, as described in a previous study [35]. Pixels with *I* ≥ 134 were classified as rice leaf and stem regions, whereas those with *I* < 134 were classified as non-rice regions.

$$I=128+128\times \frac{G-R}{G+R}$$

where *R* and *G* represent the red and green pixel intensities (0–255), respectively.

**Figure 2:**
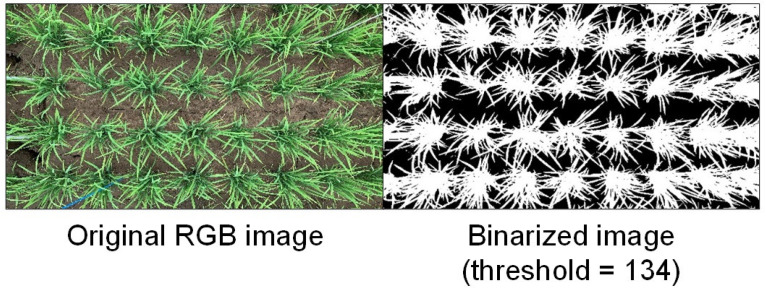
Example of leaf detection by threshold processing. The RGB canopy image (**left**) was converted to a binary image (**right**) using a threshold value of 134 to extract green pixels corresponding to rice leaves.

Figure 2 shows examples of the original RGB image and the extracted rice leaf and stem regions obtained using the above thresholding procedure. The left image shows the RGB image captured using a smartphone, and the right image shows the binarized result in which pixels satisfying *I* ≥ 134 are classified as rice leaf and stem regions. The green parts are highlighted as white, confirming that the rice canopy regions were appropriately extracted.

The canopy cover ratio (%) was calculated from the number of extracted rice leaf and stem pixels and the total number of pixels in the image. The estimated tiller number (tillers m^−2^) was then calculated using a quadratic regression model with the canopy cover ratio as the explanatory variable. In this study, the following estimation equation was adopted:

$$Tiller\;number\;(tillers\;m^{-2})\;=\;-\;0.0548\;\times \;{\left(canopy\;cover\right)}^{2}\;+\;9.13\;\times \;(canopy\;cover)\;+\;77.7$$

This estimation equation was developed based on field experimental data reported in a previous study [34] and was derived under planting densities of 11.0–18.3 hills m^−2^ for the cultivar ‘Koshihikari’. Using this model, the tiller number of an entire paddy field can be estimated from an image covering 16 hills (4 rows × 4 hills).

In this study, vegetation extraction was performed using a red–green discriminant index based on the R and G channels, while the blue channel was intentionally excluded. The blue band is highly sensitive to illumination variability and specular reflections from water surfaces under field conditions, which can introduce noise and degrade segmentation stability [37,38]. Although RGB-based vegetation indices such as Excess Green (ExG) have been widely used in previous studies [39,40], we adopted a simpler red–green ratio approach to prioritize computational efficiency, robustness, and real-time performance on smartphone devices. Preliminary tests indicated that ExG did not provide a clear accuracy advantage under variable field lighting conditions, while increasing computational complexity. Therefore, a lightweight index optimized for on-site smartphone processing was selected.

#### 2.2.3. Diagnostic Criteria (Cultivar-Specific Target Tiller Numbers)

The estimated tiller number was compared with cultivar-specific target tiller numbers defined to determine the optimal timing of midseason drainage. The target values were set based on the results of previous field experiments and the cultivation guidelines of Chiba Prefecture [17]. The target tiller numbers were defined as 400 tillers m^−2^ for ‘Fusaotome’, 360 tillers m^−2^ for ‘Fusakogane’, and 320 tillers m^−2^ for ‘Koshihikari’.

If the estimated tiller number was equal to or higher than the target value, the system diagnosed the field as being at the “optimal timing for midseason drainage”. If the estimated value was lower than the target value, the field was diagnosed as having an “insufficient tiller number”. The diagnostic result was immediately displayed on the smartphone screen, enabling rapid decision-making in practical rice production.

### 2.3. Image Acquisition Method

**Figure 3:**
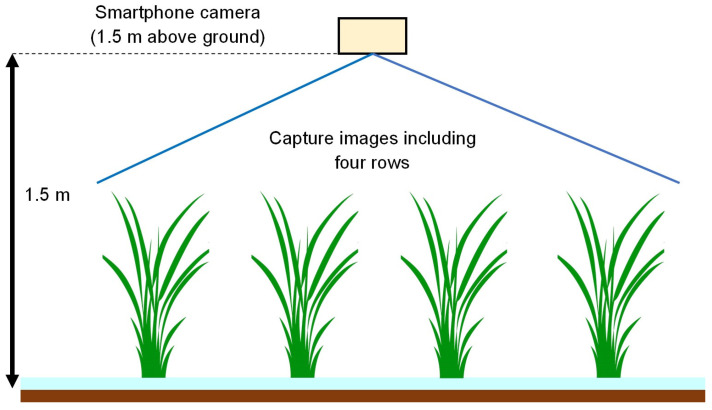
Method of capturing the rice canopy image.

In this study, rice canopy images were captured from above using a smartphone and used for image analysis. An overview of the image acquisition method is shown in Figure 3. A Xiaomi smartphone (Xiaomi Mi 11 Lite 5G) equipped with a standard built-in camera (approximately equivalent to 26 mm in 35 mm format) was used for image acquisition. Images were captured at a resolution of 4640 × 3472 pixels, and all camera settings were kept in automatic mode.

Image acquisition was performed at representative locations in each paddy field. Photographs were taken vertically from directly above the rice canopy at a height of approximately 1.5 m above ground. The field of view was fixed so that 4 rows × 4 hills (a total of 16 hills) were consistently included in the image, thereby ensuring that the previously described tiller estimation model (Section 2.2.2) could be appropriately applied.

To minimize the influence of environmental noise, images were acquired only under the following conditions: Sunny or bright cloudy weather, Image acquisition conducted between approximately 9:00 a.m. and 3:00 p.m. to avoid extremely low light conditions or strong backlighting, Areas without visible weeds or algae, Camera angles that minimized reflections from the water surface or leaf surfaces.

Although light intensity varied depending on field conditions, no quantitative control of illumination was applied. Instead, the above conditions were used to avoid extreme differences in lighting among images.

For each cultivar, image acquisition was conducted three times: approximately one month after transplanting, immediately before the start of midseason drainage, and after the initiation of midseason drainage. After image acquisition, the images were analyzed on-site using the application, and the diagnostic results were confirmed in real time.

### 2.4. Field Experiments

Field experiments were conducted in rice paddies located at three sites in Chiba Prefecture, Japan. The cultivar ‘Fusaotome’ was tested in a sandy loam paddy field in Kimitsu City, ‘Koshihikari’ in a clayey paddy field in Futtsu City, and ‘Fusakogane’ in a loam paddy field in Sodegaura City. The transplanting dates were 5 April 2024 for ‘Fusaotome’, 25 April 2024 for ‘Koshihikari’, and 2 May 2024 for ‘Fusakogane’. The planting density was identical for all cultivars at 18.2 hills m^−2^, corresponding to a row spacing of 30 cm and a hill spacing of 18 cm.

For each cultivar, field surveys were conducted three times: approximately one month after transplanting, immediately before the start of midseason drainage, and after the initiation of midseason drainage. Rice plants showing representative growth conditions within each experimental plot were selected, and images were captured vertically from above using a smartphone according to the method described in Section 2.3. At the same time, the tiller numbers of the 16 hills (4 rows × 4 hills) included in each image were manually counted. Both the estimated values obtained from the images and the observed values were collected in three replicates to confirm reproducibility.

Crop management during the field experiments followed the conventional practices adopted at each site. Midseason drainage was also conducted according to local standard management practices, with careful control to avoid excessive drying or over-wetting of the paddy fields.

The paddy soils in Chiba Prefecture, where the study sites are located, are mainly classified as Gley lowland soils, and their soil pH has been reported to generally range from slightly acidic to neutral, around pH 6 [41]. In the paddy fields targeted in this study, extremely acidic or alkaline conditions were not expected; therefore, the influence of soil pH on the estimation results was considered to be limited.

### 2.5. Data Analysis

The accuracy of the estimated tiller number was evaluated by comparing the estimated tiller number (tillers m^−2^) calculated from captured images with the observed tiller number (tillers m^−2^) obtained by manual counting of the 16 hills included in each image. Both the estimated and observed values were expressed as the mean of three replicates. The difference between the estimated and observed values was defined as the estimation error and was evaluated using the root mean square error (RMSE) and mean bias. Linear regression analysis was conducted to examine the relationship between estimated and observed tiller numbers. Data analysis was performed using Microsoft Excel. In this study, both the magnitude and direction of estimation error (overestimation or underestimation) were examined to evaluate the performance of the proposed method.

In a previous study, the tiller increase rate around the optimal timing of midseason drainage was reported to be approximately 12–24 tillers m^−2^ day^−1^ [35]. Therefore, the practical significance of the estimation errors obtained in this study was assessed in relation to this daily tiller increase rate.

## 3. Results

### 3.1. Web Application Development

**Figure 4:**
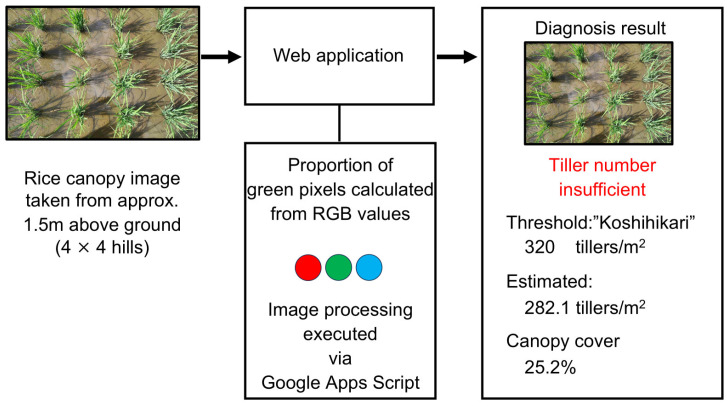
Overview of the smartphone-based diagnosis system for determining the optimal timing of midseason drainage. The system consists of image acquisition, pixel-based canopy extraction, tiller number estimation, comparison with cultivar-specific target values, and real-time diagnosis output. Colors are used for illustrative purposes only.

The midseason drainage diagnostic system developed in this study (hereafter referred to as “the proposed system”) was implemented as a web-based application that operates on a smartphone. When a user selects the target cultivar and uploads an image captured in the field, the entire process—from estimation of the tiller number to the display of the diagnostic result—is completed immediately (Figure 4).

The image analysis processes were executed on the smartphone terminal (client side) using HTML5 and JavaScript. This configuration enabled real-time processing of a series of procedures, including the extraction of rice leaf and stem regions, calculation of the canopy cover ratio, estimation of the tiller number, and comparison with the target tiller number. In contrast, Google Apps Script (GAS) was used as a lightweight serverless backend to handle part of the diagnostic decision process, thereby enabling simple system operation without the need to construct or maintain an external server.

**Figure 5:**
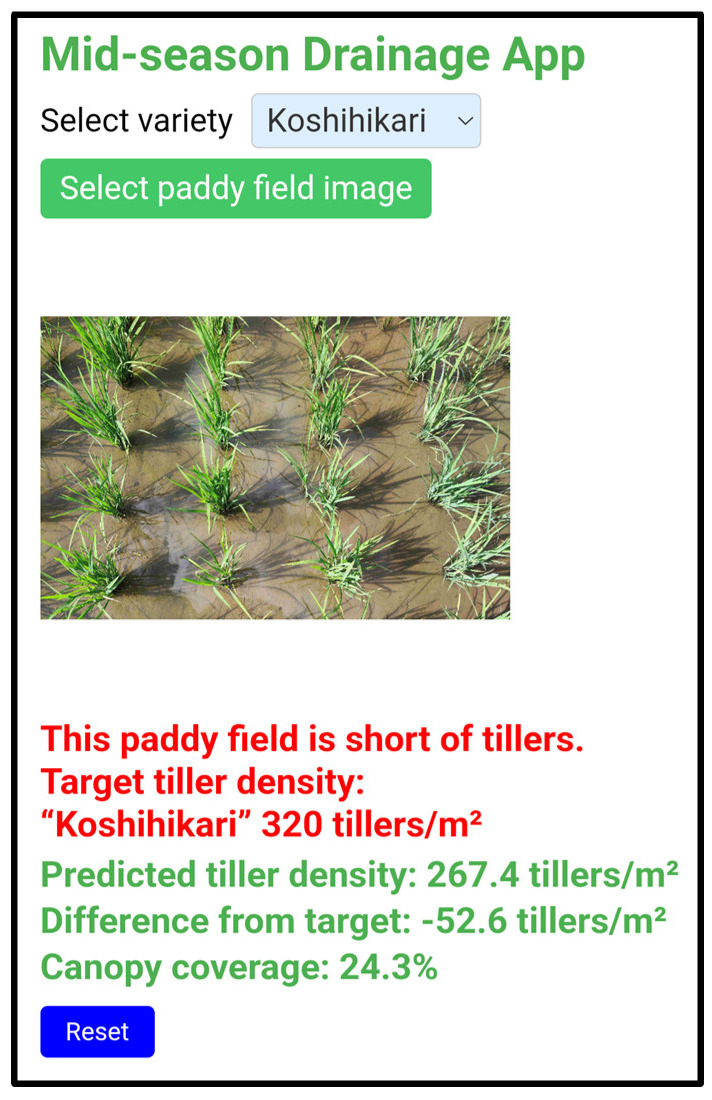
Example of the diagnosis screen of the web application. The screen displays the estimated tiller number, the cultivar-specific target tiller number, and the diagnostic result indicating whether midseason drainage is optimal.

Figure 5 shows an example of the application interface. The cultivar-specific target tiller number is automatically displayed according to the selected cultivar. When the estimated tiller number reaches the target value, the field is diagnosed as being at the “optimal timing for midseason drainage”; otherwise, it is diagnosed as having an “insufficient tiller number”. These results are displayed on the smartphone screen immediately after image acquisition, demonstrating that the proposed system enables rapid on-site decision-making for determining the timing of midseason drainage in practical rice production.

### 3.2. Results of Tiller Number Estimation

**Figure 6:**
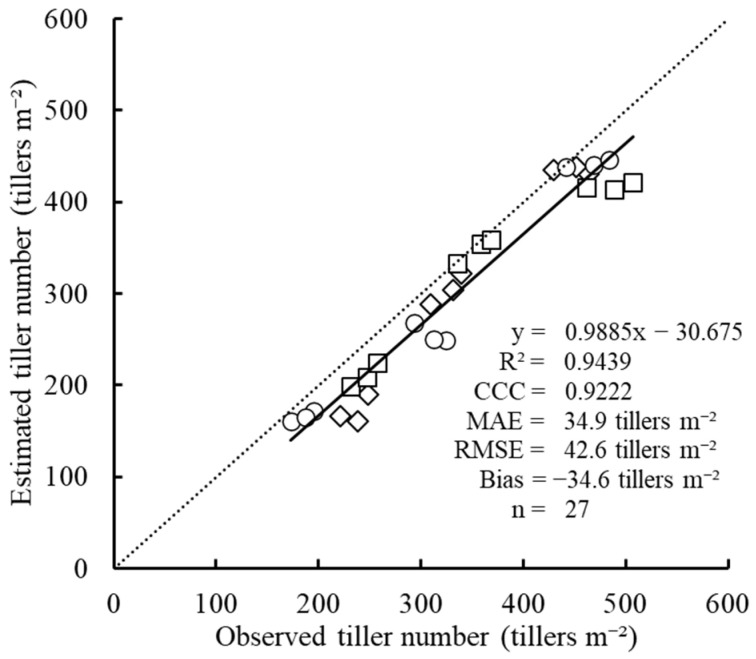
Relationship between observed and estimated tiller numbers across all cultivars, survey dates, and replicates. The dashed line represents the 1:1 line, and the solid line indicates the fitted linear regression. Symbols indicate rice cultivars: ◇, ‘Fusaotome’; □, ‘Fusakogane’; ○, ‘Koshihikari’. A strong linear relationship was observed between estimated and observed tiller numbers (R^2^ = 0.9439, n = 27). The root mean square error (RMSE) was 42.6 tillers m^−2^, and the mean absolute error (MAE) was 34.9 tillers m^−2^. Lin’s concordance correlation coefficient (CCC = 0.922) indicated strong agreement between estimated and observed values. Although a consistent negative bias (−34.6 tillers m^−2^) suggests systematic underestimation, these results demonstrate that the proposed method reliably captures both relative variation and absolute agreement in tiller number, supporting its applicability for threshold-based decision-making on midseason drainage timing.

The estimated tiller numbers calculated from the captured images increased with crop growth progression for all cultivars, showing overall trends consistent with those of the observed tiller numbers (Figure 6). However, across all cultivars and observation dates, the estimated tiller numbers tended to be lower than the observed values. This underestimation is considered to be attributable to growth stage– and canopy structure–dependent characteristics of the rice plants; specifically, when leaves tended to be more erect and inter-hill spaces remained relatively open, the visibility of basal plant parts and small tillers was insufficient in top-view images, resulting in relative discrepancies between estimated and observed values.

Overall, although the proposed system tended to underestimate the absolute tiller number, it successfully reproduced the growth stage-dependent increase patterns of tiller number with good consistency.

### 3.3. Evaluation of Estimation Accuracy

To quantitatively evaluate estimation accuracy, regression analysis was conducted using pooled data across all cultivars, survey dates, and replicates to examine the relationship between estimated and observed tiller numbers (Figure 6). A strong linear relationship was observed, with a coefficient of determination of R^2^ = 0.9439. The overall prediction error, expressed as the root mean square error (RMSE), was 42.6 tillers m^−2^, while the mean absolute error (MAE) was 34.9 tillers m^−2^. The mean bias was −34.6 tillers m^−2^, indicating a systematic tendency toward underestimation. Lin’s concordance correlation coefficient (CCC = 0.922) further demonstrated strong agreement between estimated and observed values. Despite this systematic underestimation, the high R^2^ and CCC values indicate that the proposed method reliably captures both relative variation and absolute agreement in tiller number across growth stages. Because the primary objective of this study was not precise biometric quantification but threshold-based decision support for midseason drainage timing, the observed magnitude of RMSE, MAE, and bias is considered acceptable for practical field application.

In this study, the system was evaluated as a diagnostic tool prioritizing simple field operation without requiring prior calibration or correction procedures. Therefore, offset-based bias correction using the mean bias was not applied. Nevertheless, implementing a simple bias correction approach could further improve absolute accuracy, and this will be addressed in future system refinements.

## 4. Discussion

### 4.1. Overall Estimation Performance and Practical Accuracy

Across all cultivars, survey dates, and replicates, a strong linear relationship was observed between estimated and observed tiller numbers (R^2^ = 0.9439, n = 27). In addition, Lin’s concordance correlation coefficient (CCC = 0.922) indicated high agreement between estimated and observed values, reflecting both strong correlation and low systematic deviation. The overall prediction error, expressed as the root mean square error (RMSE), was 42.6 tillers m^−2^, and the mean absolute error (MAE) was 34.9 tillers m^−2^. A consistent negative bias (−34.6 tillers m^−2^) indicated a systematic tendency toward underestimation.

Considering previously reported tiller increase rates around the midseason drainage period (approximately 12–24 tillers m^−2^ day^−1^) [35], the estimation errors obtained in this study correspond to approximately 1–3 days of growth progression. From a practical standpoint, this level of temporal uncertainty is considered acceptable for supporting threshold-based decision-making regarding midseason drainage timing.

Importantly, although the proposed system tended to underestimate absolute tiller numbers, it successfully reproduced growth stage-dependent trends with high consistency across cultivars. Because the primary objective of this study was to support relative, threshold-based field decision-making rather than precise absolute quantification, the observed levels of RMSE and bias can be regarded as practically acceptable. Moreover, although bias correction using a simple offset could potentially improve absolute accuracy, no calibration or correction procedures were applied in this study in order to prioritize operational simplicity and field usability.

### 4.2. Causes of Underestimation: Plant Architecture and Morphological Factors

One major factor contributing to the observed underestimation appears to be the influence of plant architecture during the early growth stage. In fields with a strong tendency toward deep planting, the basal parts of plants tend to close early, making small tillers difficult to visually detect in captured images. As a result, the RGB-based extraction method used in this study may have had difficulty identifying fine tillers. Such structural characteristics can lead to underestimation of canopy cover, which in turn results in underestimation of tiller number. Previous studies have reported that canopy structure and leaf arrangement significantly affect the accuracy of image-based plant diagnostics [28,29], and the trends observed in this study are consistent with these previous findings.

Furthermore, the overall underestimation tendency observed in this study is considered to be strongly influenced by the morphological characteristics of rice leaves. During the early growth stage, rice leaves tend to be more erect, and leaf overlap increases, which makes basal parts of plants and young tillers more likely to be occluded in images captured from directly above. As a result, small tillers and narrow leaves become difficult to distinguish at the pixel level, leading to omission errors in stem–leaf extraction based on RGB values. In addition, cultivar-dependent morphological differences, such as leaf width and leaf length, affect background contrast and the clarity of individual plant boundaries, thereby contributing to variability in tiller number estimation accuracy. The influence of leaf erectness, overlap, and leaf shape on image analysis accuracy has also been reported in previous studies targeting crop canopies, including rice [42]. Therefore, the underestimation tendency observed in this study can be reasonably explained from a morphological perspective.

### 4.3. Image Acquisition Strategy and Viewpoint Limitations

To prioritize rapid field diagnosis and operational simplicity, tiller number estimation was performed using single-view images captured vertically above the rice canopy. This approach offers a significant advantage in that image acquisition is simple and can be easily performed by farmers in the field. However, methods relying solely on nadir-view images cannot completely avoid occlusion effects caused by leaf overlap and shooting angle, and it has been pointed out that small tillers and basal plant parts are particularly difficult to observe under such conditions [43].

As a potential future improvement to address these issues, the combined use of multi-angle or oblique-view images can be considered. Integrating information from multiple viewpoints may reduce the effects of leaf overlap and occlusion and improve the visibility of organs located inside or in the lower layers of the canopy. Indeed, studies focusing on rice have reported that the use of multi-angle images improves the estimation accuracy of panicle number and canopy structure [44]. However, multi-angle image acquisition inevitably increases both operational complexity and computational load. Therefore, from the perspective of maintaining field-level usability, careful system design—such as the use of shooting guides or the selection of representative viewpoints—is required to balance practicality and estimation accuracy.

### 4.4. Robustness Under Weather and Environmental Variability

With regard to the applicability of the proposed system, robustness under varying weather conditions and real field environments is an important consideration. The tiller estimation method developed in this study does not directly use meteorological variables such as air temperature or solar radiation as input data. Instead, diagnosis is performed based on image information that reflects the actual growth state of the rice canopy. Consequently, the effects of extreme weather conditions, including high or low temperatures, are indirectly incorporated into the diagnostic results through changes in growth rate and canopy architecture.

Evaluations were conducted using data obtained from multiple paddy fields with different transplanting dates and locations, thereby covering rice grown under a range of weather conditions. Under all tested conditions, estimation results remained stable, and no critical errors or estimation failures were observed. Nevertheless, a detailed evaluation of estimation accuracy under record-breaking high-temperature years or abnormal weather conditions was not sufficiently conducted in this study and remains an important topic for future research.

At the same time, image-based phenotyping under real field conditions is known to be sensitive to environmental factors such as illumination variability, water surface reflection, and weed interference [45]. Previous studies have also reported that image-based plant diagnostic methods strongly depend on camera angle, lighting conditions, distance, and background, which may lead to reduced accuracy under practical field environments [46].

In this study, the influence of these factors was mitigated by standardizing shooting angle and time of day; however, these constraints represent limitations of the current system. Future improvements should incorporate calibration techniques and adaptive image processing strategies to enhance robustness under variable field conditions [47].

### 4.5. Model Generalization Across Cultivars and Sites

The reliability of the estimation accuracy was examined through condition-wise validation. In this study, datasets differing in cultivar, field site, and growth stage were used for evaluation, and these were treated as independent cultivation conditions. Such condition-based validation effectively functions as a form of cross-validation and enables assessment of estimation performance without reliance on specific conditions. As a result, estimation errors remained within a comparable range across all tested conditions, and no excessive error amplification biased toward particular cultivars or sites was observed. These findings indicate that the tiller estimation model used in this study possesses a certain level of generalization capability and reproducibility.

The regression model used in this study was developed based on data from the cultivar ‘Koshihikari’ and applied to other cultivars without cultivar-specific recalibration. This decision was based on the fact that the target cultivars (‘Fusaotome’, ‘Fusakogane’, and ‘Koshihikari’) are all japonica rice varieties widely grown in Chiba Prefecture, Japan, and share broadly similar plant architecture and growth patterns [48]. In practice, applying a single model across all cultivars did not result in systematic degradation of estimation accuracy, and a consistent linear relationship between estimated and observed tiller numbers was maintained. These results support the applicability of the proposed model—at least within the range of japonica cultivars examined in this study—for relative, threshold-based decision support for midseason drainage timing. However, the applicability of the model to cultivars with more pronounced architectural differences, including indica rice varieties, has not yet been evaluated and remains an important subject for future research.

### 4.6. Reproducibility and Practical Usability of the Proposed System

Compared with previously reported image-based tiller estimation and canopy analysis approaches, which often require multi-view imaging, controlled acquisition settings, or PC-based post-processing, the proposed system prioritizes real-time field usability by implementing a fully smartphone-based single-view workflow. While this design choice may trade some degree of absolute accuracy for simplicity, it enables rapid, low-cost, and scalable deployment in practical rice production environments.

The reproducibility of the proposed system was evaluated as high. The variation among the three image analyses obtained on the same observation date was very small, indicating that stable extraction of rice leaf and stem regions and consistent estimation of tiller number are possible even under simple image acquisition conditions. This high level of reproducibility is essential for ensuring the reliability of the tool in practical field applications. In addition, the proposed method can greatly reduce the labor required for manual tiller counting in paddy fields, which represents a significant practical advantage.

Furthermore, the proposed system shows substantial advantages in terms of usability compared with previously reported approaches [35]. Whereas previous methods required PC-based image analysis, the present system enables the entire process from image acquisition to tiller estimation and diagnosis of midseason drainage timing to be completed using only a smartphone. This allows real-time decision-making directly in the field and enables farmers to determine the timing of midseason drainage immediately on-site, which represents a major strength of this technology. The effectiveness of smartphone-based decision-support systems has also been reported for rice growth prediction and work scheduling [49], and the system developed in this study can similarly be positioned as a practical technology that supports rapid on-site decision-making. In addition, the system is expected to be useful as a simple diagnostic tool for agricultural extension officers during field inspections and for farmers managing multiple paddy fields.

### 4.7. Limitations of the Present Study

Although the proposed system performed well under the tested conditions, several limitations remain.

First, to further improve estimation accuracy, it will be necessary to standardize shooting distance and camera angle, as well as to introduce preprocessing techniques to correct fluctuations in RGB values caused by water surface reflection, shading, and leaf color variation (e.g., color space transformation and luminance correction). However, because the primary strength of the proposed system lies in its simplicity and smartphone-only operation, any additional processing must be implemented carefully to avoid impairing usability and lightweight system performance in field conditions.

Second, the extraction accuracy of small tillers remains limited under certain canopy structures. In particular, in fields where the basal parts of plants tend to close, small tillers are difficult to detect using pixel-based thresholding alone. This represents a methodological constraint of the current RGB-based segmentation approach.

Third, the present study was conducted over a single year at three experimental sites. Therefore, the robustness of the proposed model under broader climatic, regional, and varietal conditions has not yet been fully verified. Additional validation using multi-year and multi-site datasets will be required to confirm the general applicability of the system across diverse rice-growing environments.

Fourth, to focus on cultivar-dependent differences, the evaluation in this study was conducted under a unified planting density. Although the tiller estimation model was originally developed using image data obtained from fields with varying planting densities—suggesting a certain degree of robustness—the present study did not systematically evaluate model performance under explicitly different density conditions. This remains a limitation of the current experimental design.

Finally, the present evaluation was conducted using a single smartphone model to control device-related variability and ensure consistent experimental conditions. While earlier developmental versions of the system were tested across multiple smartphone platforms [33,34,35], device-dependent variability related to camera sensors, lens characteristics, and internal image-processing pipelines was not quantitatively assessed in this study.

### 4.8. Future Directions and System Improvements

The present results suggest several concrete directions for future system development. To address limitations in image-based tiller detection, future studies should explore the acquisition of higher-resolution images and the introduction of deep learning–based approaches for analyzing leaf and stem structures. The application of structural models capable of handling complex canopy architecture may improve detection performance, particularly for small or occluded tillers.

In addition, systematic validation using multi-year, multi-site, and multi-cultivar datasets will be essential to strengthen the robustness and generalizability of the proposed method under diverse environmental and agronomic conditions.

Furthermore, the current system requires users to manually adjust the field of view so that a 4 × 4 hill (16-hill) area is captured within the image, as an automatic cropping (auto-trimming) function has not yet been implemented. Future development could incorporate automatic region extraction based on shooting guides or hill arrangement detection, enabling automated cropping of the target area and improving both operational consistency and estimation accuracy.

Another important direction is systematic cross-device validation using identical field datasets. This will help clarify the influence of camera sensor characteristics and image-processing pipelines among smartphone models, and further strengthen the portability and reliability of the proposed system across different mobile platforms.

### 4.9. Implications for Smart Agriculture and Environmental Sustainability

Overall, the results of this study demonstrate that the proposed system is a sufficiently practical, simple, and rapid diagnostic tool for determining the optimal timing of midseason drainage as an alternative to conventional manual measurement. By enabling appropriate water management, the system is expected to contribute to the stabilization of rice yield and grain quality. In addition, proper implementation of midseason drainage is known to reduce methane emissions from paddy fields [13], and thus the proposed system is also a promising technology not only for promoting smart agriculture but also for contributing to the reduction in environmental burdens.

## 5. Conclusions

In this study, we developed a simple web-based application that estimates the tiller number from rice canopy images captured using a smartphone and determines the optimal timing of midseason drainage. A key feature of the proposed system is that all processes—from image acquisition to analysis and diagnosis—are completed entirely on a smartphone directly in the field.

Field experiments demonstrated that although the proposed system systematically underestimated absolute tiller numbers, the estimated values exhibited a strong linear relationship with observed values across cultivars, survey dates, and replicates (R^2^ = 0.9439). The overall prediction error, expressed as the root mean square error (RMSE), was 42.6 tillers m^−2^, and the mean bias was −34.6 tillers m^−2^, indicating a consistent negative bias. These errors correspond to approximately 1–3 days of growth progression, which is considered to fall within a practically acceptable range for determining the optimal timing of midseason drainage. Importantly, the proposed system reliably captured relative changes in tiller number across growth stages, supporting its suitability for threshold-based and timing-oriented decision-making rather than precise absolute quantification. Therefore, the system is effective as a rapid, objective, and field-ready decision-support tool.

The proposed system is expected to be useful as a simple diagnostic tool for agricultural extension activities and for farmers managing multiple paddy fields. In addition to stabilizing grain quality and yield through appropriate implementation of midseason drainage, the system also has the potential to contribute to the reduction in methane emissions from paddy fields. Therefore, the proposed technology is promising for both the promotion of smart agriculture and the mitigation of environmental impacts.

Nevertheless, further improvements in estimation accuracy are needed through the standardization of image acquisition conditions, the introduction of preprocessing techniques to correct reflections and shading, image processing approaches to compensate for structural differences at the basal parts of plants, and multi-site validation across different years, cultivars, and regions. Moreover, the integration of automatic image-trimming functions and machine learning–based analysis of leaf and stem structures is expected to enable the development of a more robust and versatile diagnostic system.

Overall, the proposed system provides a practical and user-friendly technology for estimating the optimal timing of midseason drainage. In the future, it also holds strong potential for broader application in decision support for rice cultivation, including topdressing timing and tiller management.

## Figures and Tables

**Table 1:** System requirements and analysis response time of the proposed smartphone-based diagnosis system.

Item	Specification
Device	Smartphone
OS	Android 12
Browser/App	Web browser (Chrome)
Camera	Built-in camera
Network	Required for system access; not required during image analysis
Analysis time	Approximately 5 s per image

## Data Availability

The data presented in this study are available on request from the corresponding author. The data are not publicly available due to privacy and field management restrictions.
